# The development of response surface pathway design to reduce animal numbers in toxicity studies

**DOI:** 10.1186/2050-6511-15-18

**Published:** 2014-03-25

**Authors:** Sagita Dewi, Tore Aune, John A Aasen Bunæs, Adrian J Smith, Stig Larsen

**Affiliations:** 1 Centre for Epidemiology and Biostatistics, Faculty of Veterinary Medicine and Biosciences, Norwegian University of Life Sciences, P.O. Box 5003, 1432 Ås, Norway; 2 Department of Food Safety and Infection Biology, Faculty of Veterinary Medicine and Biosciences, Norwegian University of Life Sciences, P.O. Box 5003, 1432 Ås, Norway; 3 Norecopa, c/o Norwegian Veterinary Institute, P.O. Box 750, Sentrum 0106 Oslo, Norway

**Keywords:** Response surface pathway design (RSP), Estimating LD_50_, Up and down designs, Random walk design, Reduction in animal use

## Abstract

**Background:**

This study describes the development of Response Surface Pathway (RSP) design, assesses its performance and effectiveness in estimating LD_50,_ and compares RSP with Up and Down Procedures (UDPs) and Random Walk (RW) design.

**Methods:**

A basic 4-level RSP design was used on 36 male ICR mice given intraperitoneal doses of Yessotoxin. Simulations were performed to optimise the design. A *k*-adjustment factor was introduced to ensure coverage of the dose window and calculate the dose steps. Instead of using equal numbers of mice on all levels, the number of mice was increased at each design level. Additionally, the binomial outcome variable was changed to multinomial. The performance of the RSP designs and a comparison of UDPs and RW were assessed by simulations. The optimised 4-level RSP design was used on 24 female NMRI mice given Azaspiracid-1 intraperitoneally.

**Results:**

The *in vivo* experiment with basic 4-level RSP design estimated the LD_50_ of Yessotoxin to be 463 μg/kgBW (95% CI: 383–535). By inclusion of the k-adjustment factor with equal or increasing numbers of mice on increasing dose levels, the estimate changed to 481 μg/kgBW (95% CI: 362–566) and 447 μg/kgBW (95% CI: 378–504 μg/kgBW), respectively. The optimised 4-level RSP estimated the LD_50_ to be 473 μg/kgBW (95% CI: 442–517). A similar increase in power was demonstrated using the optimised RSP design on real Azaspiracid-1 data. The simulations showed that the inclusion of the k-adjustment factor, reduction in sample size by increasing the number of mice on higher design levels and incorporation of a multinomial outcome gave estimates of the LD_50_ that were as good as those with the basic RSP design. Furthermore, optimised RSP design performed on just three levels reduced the number of animals from 36 to 15 without loss of information, when compared with the 4-level designs. Simulated comparison of the RSP design with UDPs and RW design demonstrated the superiority of RSP.

**Conclusion:**

Optimised RSP design reduces the number of animals needed. The design converges rapidly on the area of interest and is at least as efficient as both the UDPs and RW design.

## Background

In recent years, acute toxicity studies have been refined to improve animal welfare. In particular, LD_50_ studies are no longer the method of choice in toxicology studies associated with pharmaceutical drug development, or the development of food additives, flavourings, contact materials and cosmetics [1]. In some areas, however, LD_50_ studies cannot yet be replaced by other methods. There is an urgent need for alternative methods, both for ethical reasons and because of the shortcomings of the bioassays, in particular their sensitivity and specificity. *In vitro* methods have been recommended by international expert groups [2]. However, before these can be adopted information on the relative toxicities of all relevant analogues in each toxin group must be obtained, in order to establish toxic equivalency factors (TEFs) [3]. The TEF approach was initially developed to estimate the potential toxicity of mixtures of dioxins, dibenzofurans and PCBs [4-6]. TEF studies of marine algal toxins are based on LD_50_ studies in mice [3]. Ideally, these studies should be performed by exposure via the oral route. However, due to the scarcity of pure toxins in sufficient quantities, LD_50_ studies are performed by intraperitoneal (IP) injections in mice [7]. Until laboratory animal studies can be replaced completely, it is of the utmost importance to optimise trial design, in order to reduce the number of animals used for this purpose [8].

The classical LD_50_ design introduced by Trevan in 1927 requires the use of a large number of animals [9]. The design has been criticised for both ethical and scientific reasons [10,11]. The Organization for Economic Cooperation and Development (OECD) removed the classical LD_50_ (OECD 401) from their acute oral toxicity guidelines in 2002 [12] and recommended OECD 425, the Up and Down Procedure (UDP) to optimise LD_50_ design [13]. The UDP is a sequential procedure that results in rapid convergence on the area of interest, the dose for each animal being adjusted up or down depending on the outcome for the previous animal. This approach was established in 1948 [14] and Bruce proposed the use of the UDP for determination of acute toxicity to chemicals [15]. The UDP in the OECD guidelines uses a calculated dose progression factor based on the antilog of 1 divided by the estimated slope of the dose–response curve and should stay constant during testing. Single animals are dosed until one of three criteria to stop the trial is met [13].

The OECD’s UDP can only estimate the point of interest and cannot be used to construct a dose–response curve [9]. Furthermore, the single-animal strategy does not take into account inherent biological variation, or the practical problems that arise from having to test each mouse individually. Tsutakawa introduced a block up and down method known as Random Walk (RW) [16], where every sequence used more than one mouse. This design reduces the number of trials and adds flexibility. The experiment starts with *n* observations on a given level *d*_*j*_ and continues with *n* observations, with the dose determined by a procedure for generating the sequences.

In the field of engineering, the term Response Surface is commonly used to obtain the optimal value for one response variable. By combining the philosophy of UDP and the principles of Response Surface methodology [17] the basic Response Surface Pathway (RSP) design was created.

**Figure 1:**
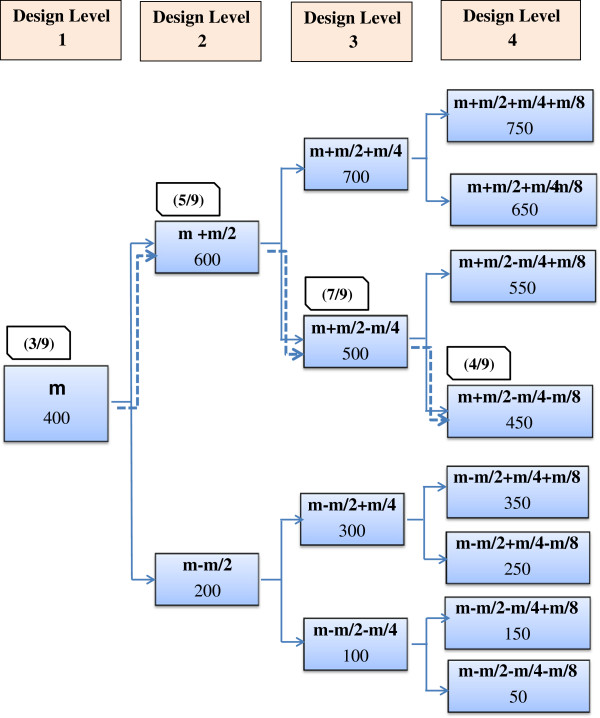
**The pathway obtained in the *in vivo *Yessotoxin study using the basic 4-level RSP design.** The basic 4-level RSP design in *in vivo* Yessotoxin study used 9 mice on each design level with the binomial outcome. The pathway obtained is indicated by dashed line. The doses are given in μg/kg BW and the numbers given in brackets are the ratios of dead mice.

Basic RSP design is based on the theory of stochastic chain models, where the procedure for generating the next step is based on the response at the previous one. The design consists of *n* levels in which the results obtained on one design level determine the dose to be used on the next. The starting dose used on the first design level is designated *m*. This dose may be an educated guess made by the responsible toxicologist. If a dose window for the given toxin is known, the mid-dose of this window might be a suitable choice. In order to estimate the LD_50_, an odd number of laboratory animals is assigned to this starting dose. If more than 50% of the animals die, the dose to be used on the second design level is reduced by *m/2*. If not, the dose is increased by the same amount (Figure 1). The same number of animals is included on the second design level. If 50% die, the second design level dose is reduced by *m/4* or conversely, if the animals survive, increased by *m/4* on the third design level. In general, the dose to be used on design level *i* will be given by the equation:

(1) $$\begin{array}{ll} m_{i}=m_{i-1}\pm \frac{m}{2^{i-1}} & \mathrm{where}\;m_{i-1}\;\mathrm{denotes}\;\mathrm{the}\;\mathrm{dose}\;\mathrm{used}\\ & \mathrm{on}\;\mathrm{design}\;\mathrm{level}\;i-1. \end{array}$$

This basic RSP-design has previously been used in toxicity studies to estimate LD_50_ with satisfactory results [18,19]. However, these studies used a large amount of animals and did not describe in detail the rationale behind the dose steps. Using the data from one of these studies [19], the concept of RSP has been refined and developed using simulation procedures.

The aims of this paper were to introduce and describe the optimisation of RSP design, to assess its performance and effectiveness in estimation of LD_50_ and to compare it with UDPs and RW design using simulations performed on data from an *in vivo* experiment.

## Methods

### Material

*The first in vivo study* has been described in detail previously [19], and comprised 36 male ICR mice of SPF quality (16-20 g) divided into 4 equal groups. The study was conducted in 2006 and was authorized by the Norwegian Animal Research Authority, in accordance with the Norwegian Regulation on Animal Experimentation. The mice were given Yessotoxin (YTX) IP at varying concentrations with a predefined LD_50_ dose window of 100–700 μg/kg body weight (BW). The outcome variable was “death” within the 24 hours after injection.

*Simulation study*: All the simulation studies used the LD_50_ of YTX derived from the material in the *in-vivo* study to estimate the probability of death at a given dose.

*The second in vivo study* consisted of 24 female NMRI mice of SPF quality (15–21 g), randomly divided into 4 groups comprising 3, 5, 7 and 9 mice per cage, respectively. The mice were supplied 1 week before the experiment for acclimatisation purposes. They were housed on aspen bedding in standard macrolone cages on a 12:12 light/dark cycle at 21-23°C with food and water available *ad libitum*. The mice were nulliparous and were not fasted before treatment. Injection volumes were adjusted for bodyweight to 1 mL/20 g BW. The animals were observed for 24 hours after injection. Symptoms and time to death were recorded. The study was conducted in 2011 with the authorization of the Norwegian Animal Research Authority, application number FOTS ID: 1132), in accordance with the Norwegian Regulation on Animal Experimentation. The mice were given Azaspiracid-1 (AZA1) IP in four different concentrations with a predefined LD_50_ dose window of 25 - 375 μg/kg BW. The outcome variable was the number of dead mice within 24 hours after injection.

### Optimisation of the basic RSP design

Some prior information about the toxicity range is always available, and can also be obtained by educated guesswork. The study can then be focused on the area of interest, thereby increasing the amount of useful information and the efficiency of animal use. In basic RSP design, no attention is paid to upper or lower doses.

Let *D*_*U*_ and *D*_*L*_ denote the upper and the lower limits of the dose window, respectively.

Let *m* denote the mid dose of the dose window, *m*_*i*_ the dose on design level *i* and *k* the dose adjustment factor. The dose on the design level *i* is then given by the equation

(2) $$\begin{array}{ll} m_{i}=m_{i-1}\pm \frac{m}{k^{i-1}}; & m_{i-1}\mathrm{denotes}\;\mathrm{the}\;\mathrm{dose}\;\mathrm{used}\\ & \mathrm{on}\;\mathrm{design}\;\mathrm{level}\;i-1 \end{array}$$

Using the formula for the sum of a geometric series [20,21], the upper dose *D*_*U*_ of the window on the highest design level *n* will be given by:

(3) $$D_{U}=m\frac{\left(k^{n}-1\right)}{\left(k^{n}-k^{n-1}\right)}$$

The dose window of the LD_50_ for YTX was from *D*_*L*_ = 100 μg/kg BW to *D*_*U*_ = 700 μg/kg BW. The middle dose of the window was used as the starting dose, i.e. *m* = 400 μg/kg BW. Using equation (3) above, the adjustment factor *k* was:

$$700=400\frac{\left(k^{4}-1\right)}{\left(k^{4}-k^{3}\right)}\to k=2.21$$

For AZA1, the predefined dose window of the LD_50_ was from *D*_*L*_ = 25 μg/kg BW to *D*_*U*_ = 375 μg/kg BW which gave a starting dose of 200 μg/kg BW. Using equation (3) above this gives an adjustment factor *k* of:

$$375=200\frac{\left(k^{4}-1\right)}{\left(k^{4}-k^{3}\right)}\to k=2.0$$

#### Increasing the number of animals with increased design level

The basic RSP design uses an equal number of animals on each design level. The starting dose is either based on an educated guess or on the mid-dose of the dose window. Due to the structure of the design, it is likely to approach the parameter in question with increasing design level. It is therefore unnecessary to use the same number of animals at the starting dose as on the highest design level. By using the lowest possible number of animals at the starting dose and increasing the number at increasing design levels, the total number of animals needed will be reduced without loss of information. One practical solution is to use three animals on the first level, five on the second, seven on the third, nine on the fourth, and so on.

#### Multinomial outcome variable

In toxicological studies the most common outcome variable is binomial. In the determination of the LD_50_, the outcome is "dead" or "alive" after a certain time interval. The decision to either increase or decrease the dose on the next design level is based upon whether there were more or less than 50% dead animals on the previous level. However, there is clearly a difference between outcomes where 0, 1, 2 or 3 animals die. It is reasonable to assume that by changing the outcome variable from binomial to multinomial, the amount of information obtained will increase. The binomial outcome variable can be replaced by "the number of dead animals". If none of the animals die, the toxin dose can be increased maximally on the next level. Conversely, if all the animals die on a given level, an equally large decrease in the dose is performed (Table 1).

**Table 1:** **The doses assigned on the four design levels using the k-adjustment factor and the dose m**_**i **_**at level *i***

**Design level I (3 animals)**	**Design level 2 (5 animals)**		**Design level 3 (7 animals)**		**Design level 4 (9 animals)**	
**Outcome**	**Dose (m**_**2**_**)**	**Outcome**	**Dose (m**_**3**_**)**	**Outcome**	**Dose (m**_**4**_**)**	**Outcome**
0	m + m/k	0	m_2_ + m/k^2^	0	m_3_ + m/k^3^	0
1	m + m/k^2^	1	m_2_ + m/k^3^	1	m_3_ + m/k^4^	1
2	m - m/k^2^	2	m_2_ + m/k^4^	2	m_3_ + m/k^5^	2
3	m - m/k	3	m_2_ - m/k^4^	3	m_3_ + m/k^6^	3
		4	m_2_ - m/k^3^	4	m_3_ - m/k^6^	4
		5	m_2_ - m/k^2^	5	m_3_ - m/k^5^	5
				6	m_3_ - m/k^4^	6
				7	m_3_ - m/k^3^	7
						8
						9

The dose m = m_1_ is the starting dose. The number of mice used on the design levels was 3, 5, 7 and 9, respectively.

### Comparisons of 3-level RSP design, UDPs and RW design

Simulation of all the four designs was performed on the first *in vivo* material. The 3-level RSP with multinomial outcome was performed with 3, 5, and 7 mice on design levels 1, 2, and 3 respectively. The *k*-adjustment factor in the 3-level situation was calculated to be 2. The UDP simulation was performed using OECD 425 guidelines [13]. The LD_50_ of YTX was assumed to be 400 μg/kg BW with a sigma of 0.25 μg/kg BW and a progression factor of 1.78. The same dose sequence and starting dose for OECD’s UDP was used in both simple UDP and RW design, which use 3 mice on each level. The dose was decreased if more than 50% mice died, and increased if less than 50% mice died. If no mice died in the RW design, the dose was increased. If more than 2 mice died, the dose was reduced. If one mouse died, a coin was tossed to decide whether to increase the dose or stay at the same dose. The starting dose was 225 μg/kg BW and the simulations were halted when a total of 15 animals had been included.

### Simulation procedure

The YTX *in vivo* study was used to estimate the probability of death at a given dose using logistic regression in a binomial distribution. A total of 10,000 simulated mice samples were generated for each dose. The simulation procedure was repeated 10 times and the average of the 10 outcomes was used as the simulated result. The same procedure was also carried out to compare 3-level RSP Design, UDPs and RW Design. Three pathways within the RSP design were considered to assess its performance, with binomial outcome, k-adjustment factor and either the same number of animals at each design level or increasing numbers at increasing design levels, respectively.

For the optimised 4-level RSP design with multinomial outcome, k-adjustment factor and increasing numbers of mice at increasing design levels, eighteen pathways were created, but only four are of interest.

When assessing the performance of optimised 3-level RSP design with multinomial outcome, k-adjustment factor and increasing number of mice with increasing design level, eight pathways were created. As in the previous simulations, the probability of dead mice at a given dose was estimated using logistic regression in a binomial distribution from the YTX *in vivo* study. For each scenario, the mean number of dead mice at the assigned dose in 10,000 simulations was recorded and the outcome was used to simulate the number of dead mice at each design level.

### Statistical analysis

The results are expressed with 95% confidence intervals (CIs) using isotonic regression [22] and trimmed Spearman-Karber regression estimation [23]. The isotonic regression was performed using the pooled adjacent-violators algorithm (PAVA) [22]. The CIs were obtained using parametric bootstrap numerical methods [24]. The trimmed Spearman-Karber program originated from Montana State University and was modified at the Duluth and Athens National Exposure Research Laboratories [25]. The LD_50_ in OECD’s UDP was analysed by Maximum Likelihood Estimation (MLE) using AOT425 software [26].

## Results

### Basic RSP design (*in vivo* YTX study)

Three of the nine mice at the starting dose of 400 μg/kg BW died and the dose for the second design level was increased to 600 μg/kg BW (Figure 1). This resulted in the death of five of nine mice, which resulted in a dose of 500 μg/kg BW on the third design level. On this design level, seven of nine mice died and the dose for the fourth design was decreased to 450 μg/kg BW. Four of nine mice died on the fourth and last design level. Based on the data obtained, the LD_50_ of Yessotoxin was estimated to be 463 μg/kg BW (Table 2).

**Table 2:** **The estimated LD**_**50 **_**of Yessotoxin (YTX) in different developmental stages of RSP design**

**Design**	**Dose (μg/kg BW)**	**Proportion of dead mice**	**LD**_**50 **_**with 95% CI (μg/kg BW)**	
			**Isotonic regression**	**Spearman-Karber**
Basic RSP	400	3/9 (0.33)	463 (383 – 535)	457 (400 – 522)
	450	4/9 (0.44)		
	500	7/9 (0.78)		
	600	5/9 (0.56)		
*) Included *k*-adjustment factor	400	3/9 (0.33)	481 (362 – 566)	480 (408 – 565)
	462	4/9 (0.44)		
	499	5/9 (0.56)		
	581	5/9 (0.56)		
*) Optimising use of mice	400	1/3 (0.33)	447 (378 – 504)	444 (379 – 521)
	462	5/9 (0.56)		
	499	5/7 (0.71)		
	581	4/5 (0.80)		
*) Multinomial decision variable with all four design levels	400	1/3 (0.33)	473 (442 – 517)	471 (430 – 516)
	465	3/7 (0.43)		
	468	4/9 (0.44)		
	482	3/5 (0.60)		

The results are expressed with 95% Confidence Intervals. *) Simulated data based on the 36 observations in the basic RSP model.

The ways in which the pathways and results from a single simulation study were used in each of the RSP designs to estimate the LD_50_ are described below:

### Application of the *k-*adjustment factor (simulated YTX study)

**Figure 2:**
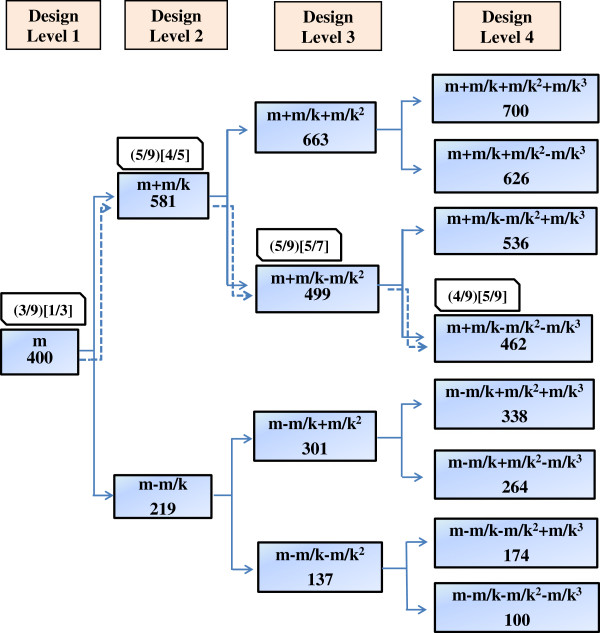
**Simulated Yessotoxin studies using 4-level RSP designs with k-adjustment factor with fixed and increasing number of mice.** The simulated Yessotoxin pathways obtained in the two 4-level RSP designs. The pathways obtained are shown by dashed line. The doses are given in μg/kg BW. The first design incorporates binomial outcome, k-adjustment factor, and 9 mice at each design level, where the ratios of dead mice are given in bracket. The second design incorporates binomial outcome, k-adjustment factor and increasing number of mice from 3 at the first level to 9 at the fourth, where the ratios of dead mice are given in square brackets [ ].

The *k*-adjustment factor of 2.21 was calculated to ensure that the design covered the total predefined dose window (Figure 2). Of the nine simulated mice given 400 μg/kg BW, three died and the dose for the second design level was increased to 581 μg/kg BW. On this design level, five mice died and the dose for the third design level was decreased to 499 μg/kg BW. The simulated results on this design level again resulted in five dead mice and the dose for the fourth and last design level was set to 462 μg/kg BW. Four of the nine mice died. Based on these simulated data, the LD_50_ was estimated to be 481 μg/kg BW (Table 2).

### Increasing number of mice with increasing design level (simulated YTX study)

Of the three mice assigned to the first design level given 400 μg/kg BW, one died (Figure 2). The dose for the second design level was increased to 581 μg/kg BW and five mice were included. Four of these five died and the dose for the next design level was reduced to 499 μg/kg BW. Of the seven mice on this third design level, five died, which led to a reduction to 462 μg/kg BW for the fourth design level. Nine mice were simulated on this level and 5 died. Based on these simulated data, the LD_50_ was estimated to be 447 μg/kg BW (Table 2).

### Number of dead mice as outcome or decision variable (simulated YTX study)

**Figure 3:**
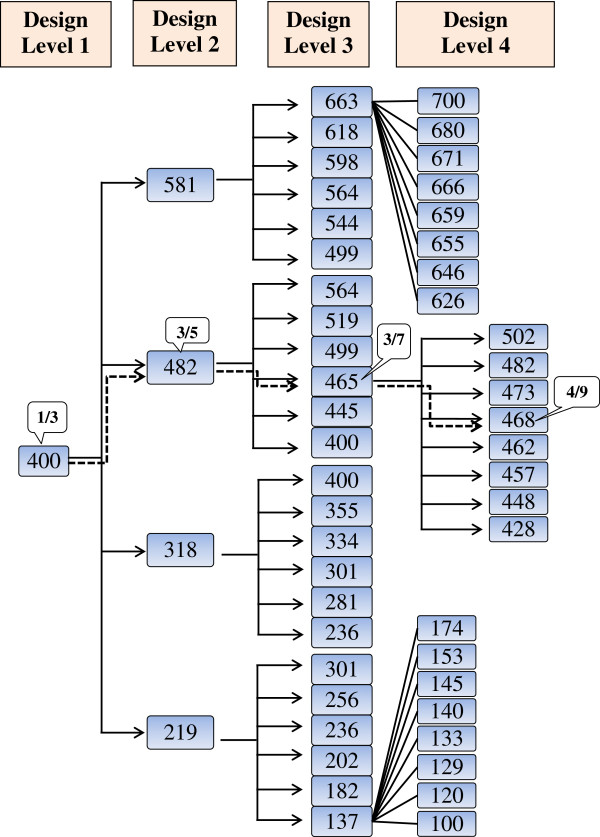
**The obtained pathway in the simulated Yessotoxin study using the optimised 4-level RSP design.** The optimised 4-level RSP design incorporates multinomial outcome, used k-adjustment factor and increasing number of mice with increasing design levels, and the pathway obtained in the simulation study is shown by dashed line. The doses are given in μg/kg BW and the numbers in the callouts represent the ratios of dead mice.

Three mice were assigned in the simulation to 400 μg/kg BW on the first design level and one died (Figure 3). The dose for the second design level was increased to 482 μg/kg BW and three of the five mice died. Consequently, the dose was decreased to 465 μg/kg BW and three of the seven mice died. The dose for the fourth design level was increased to 468 μg/kg BW and four of nine mice died. Based on the simulated data, the LD_50_ was estimated to be 473 μg/kg BW **(**Table 2).

### Performance of the RSP designs

The estimated LD_50_ of YTX with a 95% confidence interval obtained *in vitro* was used as the reference in evaluation of the simulated results of the three RSP designs.

**Figure 4:**
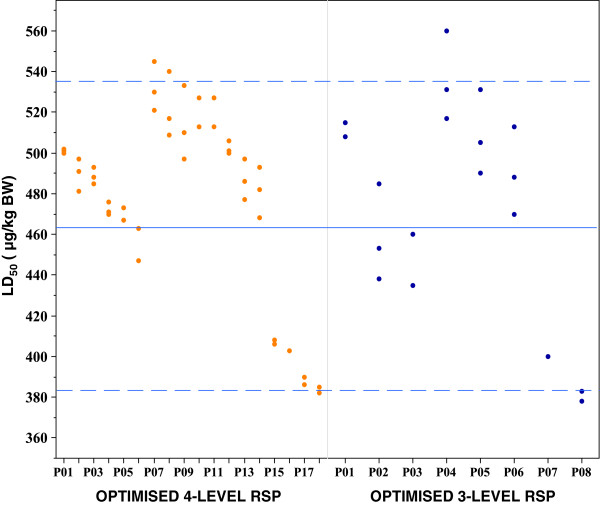
**The estimation of LD**_**50 **_**using the optimised 3- and 4-level RSP designs.** The optimised 3-level and 4-level RSP designs incorporate multinomial outcome, using k-adjustment factor and increasing number of mice with increasing design levels. The numbers of pathways are given along the x-axis, where the orange dots represent estimated value for 4-level RSP design and blue dots estimated value for 3-level RSP design.

For pathway 01 (Table 3), 24 and 10 scenarios, respectively were created for the 4-level RSP designs with binomial outcome, k-adjustment factor and nine mice on each design level or with increasing number of mice from three on the first level to nine on the fourth. For both of these two RSP designs, all the estimated LD_50_ deviated with a maximum of 10% from the *in vitro* reference. The estimated LD_50_ from pathway 02 was higher than the reference value. For the RSP design with binomial outcome, k-adjustment factor and nine mice at each level, three of the 12 scenarios deviated with a maximum of 10% from the *in vitro* reference. By increasing the number of mice from three on the first design level to nine on the fourth, two of six scenarios fulfilled the same demands. All the scenarios in pathway 03 estimated the LD_50_ to be below the *in vitro* reference value. Simulation of the optimised 4-level RSP design with multinomial outcome, k-adjustment factor and increasing numbers of mice from three to nine on the last level, estimated the LD_50_ in 11 of 18 pathways with a deviation of at most 10% from the *in vitro* reference (Figure 4).

**Table 3:** **Mean values of estimated LD**_**50 **_**of Yessotoxin in different pathways using 4-level RSP designs**

**Pathway**	**Dose on design level 1**	**Dose on design level 2**	**Dose on design level 3**	**Dose on design level 4**	**Mean LD50 (μg/kgBW)**	**95% CI of Mean LD50 (μg/kgBW)**
**4-Level RSP design with binomial outcome, k-adjustment factor and 9 animals at each design level**						
01	400 M = 3.29	581 M = 6.23	499 M = 5.01	462 M = 4.38	457	449 – 466
02	400 M = 3.29	581 M = 6.23	499 M = 5.01	536 M = 5.07	513	510 – 516
03	400 M = 3.29	219 M = 0	301 M = 1.05	338 M = 1.97	391	389 – 392
**4-Level RSP design with binomial outcome, k-adjustment factor and increasing number of mice with increasing design levels**						
01	400 M = 1.10	581 M = 3.35	499 M = 3.9	462 M = 4.41	464	449 – 479
02	400 M = 1.10	581 M = 3.35	499 M = 3.9	536 M = 5.57	510	501 – 520
03	400 M = 1.10	219 M = 0	301 M = 0.82	338 M = 1.96	379	374 – 384
**4-Level RSP design with multinomial outcome, k-adjustment factor and increasing number of mice with increasing design levels**						
01	400 M = 1.10	482 M = 2.45	499 M = 3.87	502 M = 5.06	501	498 – 503
02	400 M = 1.10	482 M = 2.45	499 M = 3.87	496 M = 4.97	490	470 – 510
03	400 M = 1.10	482 M = 2.45	465 M = 3.48	468 M = 4.51	470	432 – 508
04	400 M = 1.10	482 M = 2.45	465 M = 3.48	462 M = 4.41	455	353 – 557

The results are expressed with 95% Confidence interval. M represents the mean of dead mice based on 10,000 simulations on the assigned dose.

Comparison of the optimised 3- and 4-level RSP design resulted in similar estimation of LD_50_ (Figure 4). A total of eight pathways were formed with the optimised 3-level RSP design and the estimated LD_50_ deviated with a maximum of 10% from the *in vitro* reference in 50% of the cases.

### Application of the complete RSP design (*in vivo* AZA1 study)

**Figure 5:**
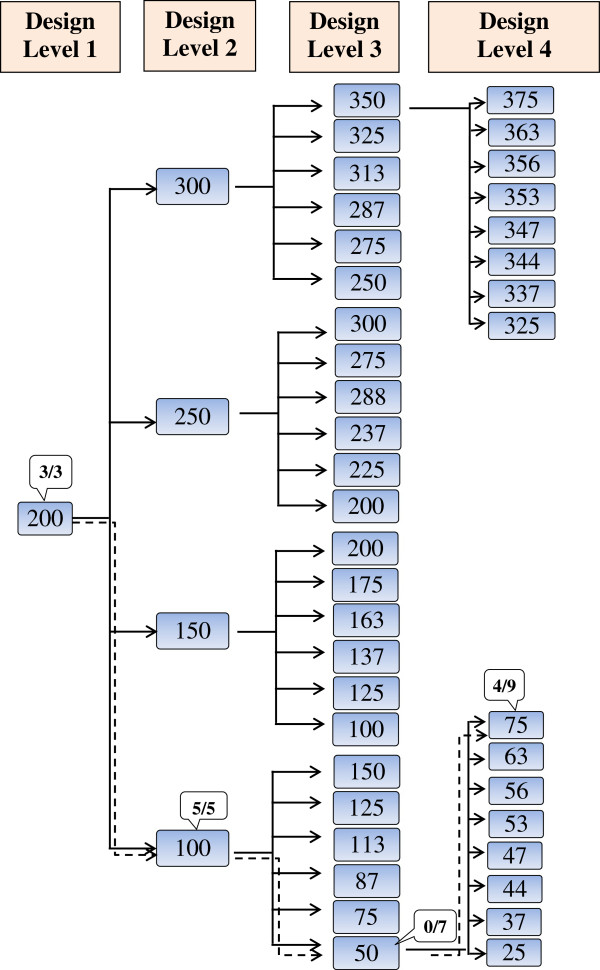
**The obtained pathway in the *in-vivo *Azaspiracid-1 study using the optimised 4-level RSP design.** The optimised 4-level RSP design incorporates multinomial outcome, using k-adjustment factor and increasing number of mice with increasing design levels, the pathway obtained is shown by dashed line. The doses are given in μg/kg BW and the numbers in the callouts represent the ratios of dead mice.

Three mice were given the starting dose of 200 μg/kg BW and all died within 24 hours (Figure 5). In accordance with the design, the dose for the second design level was decreased to 100 μg/kg BW, but all five mice died. The dose for the third design level was decreased to 50 μg/kg and all seven mice survived 24 hours after the injection. By only including data up to the third level, the LD_50_ of AZA-1 was estimated to be 76.3 μg/kg BW and 70.7 μg/kg BW using isotonic regression and the Spearman-Karber method, respectively. For the fourth design level, the dose was increased to 75 μg/kg BW, where four of the nine mice died. Inclusion of all four design levels resulted in estimation of the LD_50_ of AZA1 to be 77.5 μg/kg BW (95% CI: 68 – 88 μg/kg BW) using isotonic regression and 74 μg/kg BW (95% confidence interval: 66 – 83 μg/kg BW) using the Spearman-Karber method.

### Comparisons of 3-level RSP, UDPs and RW design

In the 3-level RSP design, three mice were given a starting dose of 400 μg/kg BW (Table 4). One of the three mice died and 500 μg/kg BW was then given to 5 mice. Three of the 5 mice died and the 7 mice on the third design level were given 475 μg/kg BW. Four of the seven mice died and the LD_50_ of YTX was estimated to be 453 μg/kg BW. The length of the CI was 133 μg/kg BW.

**Table 4:** **Comparisons of optimised 3-level RSP Design, UDPs and Random Walk Design in estimation of LD**_**50 **_**of YTX**

**Design**	**Dose (μg/kg BW)**	**Proportion of dead mice**	**LD**_**50 **_**with 95% CI (μg/kg BW)**
Multinomial outcome variable with 3-level RSP design	400	1/3	453 (380 – 513)
	475	4/7	
	500	3/5	
UDP of OECD	225	0/1	400 (198 – 977)
	400	0/3	
	710	2/2	
Simple UDP	225	0/6	400 (65.2 – 580)
	400	3/6	
	710	2/3	
Random walk design	225	0/6	400 (65.2 – 580)
	400	3/6	
	710	2/3	

The results are expressed with 95% Confidence Intervals.

The simulation of OECD’s UDP was started by including one mouse given a dose of 225 μg/kg BW. The mouse survived. The next mouse was assigned to 400 μg/kg BW and survived. The third mouse died at 710 μg/kg BW and the dose was then decreased again to 400 μg/kg BW. The fourth mouse survived this dose and the fifth mouse was given a dose of 710 μg/kg BW. The fifth mouse died and the dose for the next mouse was decreased again to 400 μg/kg BW. This sixth mouse survived. The study was stopped after six mice since one criterion for this had been met and the LD_50_ was estimated to be 400 μg/kg BW (Table 4). The length of the CI was 779 μg/kg BW ***.***

In the simple UDP, none of three mice given a starting dose 225 μg/kg BW died. In the second simulation sequence a dose of 400 μg/kg BW was given and 2 of 3 mice died. The dose was decreased to 225 μg/kg BW and all the mice survived. Consequently, a dose of 400 μg/kg BW was again assigned and one of 3 mice died. The dose was then increased to 710 μg/kg BW and 2 of 3 mice died.

In the RW design, three mice were given a dose of 225 μg/kg BW and all survived. In the next sequence, three mice were allocated a dose of 400 μg/kg BW and two mice died. The dose was decreased again, and three mice survived at 225 μg/kg BW. The dose was again increased to 400 μg/kg BW and one of 3 mice died. A coin was tossed to decide whether to increase or stay at the same dose: the dose was increased to 710 μg/kg BW and 2 of 3 mice died. The LD_50_ of YTX was estimated to be 400 μg/kg BW both in the simple UDP and RW design (Table 4), and the length of the CI was 514.8 μg/kg BW.

Comparison of the four designs showed that OECD’s UDP, simple UDP, and RW design resulted in CIs that were 4.9, 2.9, and 2.9 times larger, respectively, than the RSP design.

## Discussion

When designing a classical LD_50_ study, the dose window is divided into fixed dose intervals of equal range that cover the dose window. In the present example with a dose window of 100–700 μg/kg BW, the classical LD_50_ study with four dose levels would entail the same number of animals being assigned to doses of 100, 300, 500, and 700 μg/kg BW, respectively. A comparison of the classical LD_50_ design and RSP design demonstrates the strength of RSP. Only one of the doses chosen for a classical LD_50_ study is close to the real LD_50._ In the present study where the LD_50_ was estimated from the basic RSP design and the four simulated studies using optimised RSP design, the dose assignments ranged from 400 to 581 μg/kg BW. The lowest dose assigned, which was in the simulation of 4-level RSP design with k-adjustment factor, a fixed number of mice on each design level or increasing numbers with increasing design level, was 218 μg/kg BW. However the probability for assigning this dose in real life is minimal. Assigning doses based on the number of dead animals, as was done in the optimised RSP design, gave an opportunity to allocate a more appropriate dose.

To our knowledge, no rational procedure for choosing the size of the increase or decrease in dose has been described for UDP and the change in dose is the same regardless of the dose level. OECD recommends that the dose adjustment or progression factor in UDP design is based on the estimated slope of the dose–response curve and should remain constant throughout the test. If no information about this slope is available, a constant adjustment factor of 3.2 should be used [13]. In the basic RSP design an attempt is made to solve this problem by using a fixed but chosen dose adjustment factor of 2. Moreover, the size of the increase and decrease in dose depends on the design level. Toxicologists have quite often a predefined dose window for the toxin to be investigated. By using a chosen dose adjustment factor or predefined incremental procedure, doses outside the dose window are less likely to be included. This may reduce the amount of information in the basic RSP design and result in failures. In order to increase the power of the basic RSP design, the fixed dose adjustment factor of 2 has therefore been amended in our study to an adjustment factor *k* dependent upon the predefined dose window. This factor is related to the number of levels in the design and is calculated by a simple procedure to ensure that all the values in the dose window will be covered. Intuitively, this will increase the power of the design, even though this was not clearly demonstrated by the results of the simulations in the present study.

The choice of the middle of the dose window as a starting point is based on an assumption that this dose is more likely to be the LD_50_. Using the mid-dose and the procedure for calculation of the *k*-adjustment ensures both the upper limit (D_U_) and the lower limit *(D*_*L*_*)* of the dose window will be covered. The predefined dose window for AZA toxin was 25–375 μg/kg BW. No information was available on where in the window the value of LD_50_ was likely to be. The LD_50_ was assumed to be 200 μg/kg BW. However, the true LD_50_ turned out to be close to the lower part of the dose window. The design made a pathway directly to the area of interest after inclusion of only 3 animals.

The number of animals used in these studies was quite small. Maximum Likelihood Estimation (MLE) does not seem to be suitable in design analysis when the sample size is small [27]. The Trimmed Spearman-Karber method [23], which was used in the present study, requires at least one mortality rate that is less than or equal to 50% and at least one that is greater or equal to 50%. This method is recommended because it is freely available and it is a simple program for those who are not highly skilled in statistical analysis. Non-parametric modified isotonic regression may be a more optimal method and is recommended for small samples [22]. It is slightly more complicated, but can easily be calculated manually. Furthermore, this method assumes that the chance of toxicity does not decrease over the set of possible dosages. The analysis of the present data gave similar results with these two statistical methods.

A reduction in the number of laboratory animals is a central aim of UDPs, RW and RSP design. The UDP [15] actually recommends that only one animal is used on each dose level. The single-animal strategy used in OECD’s UDP might at first sight appear advantageous in reducing the number of animals in the study, but it does not take into account the inherent biological variation in an animal model. The probability of changing the dose on the next level in the wrong direction is high and may affect the study. Simple UDP and RW address this problem by including more than one animal on each level. As in the simple UDP and RW design, basic RSP-design uses more than one animal, with equal numbers on each dose level. Due to the structure of the design, the dose always converges on the end result with increasing dose level. It seems therefore apparent that it is unnecessary to use the same number of animals at the starting dose as on the last dose level. By using the lowest possible number of animals at the starting dose and increasing the number of animals on increasing dose levels, the total number of animals will be reduced. In order to avoid the problem of biological variation that is not addressed in OECD’s UDP, the procedure should be to start with 3 animals at the first dose level and continue with 5, 7 and 9 animals, respectively, on subsequent levels. This reduces the total number of animals from 36 to 24. The present simulation indicated that the ability of the modified RSP designs to estimate LD_50_ values was equally good as the basic RSP design.

A large disadvantage of most of the designs that have been proposed for this area is the binomial outcome variable. So far, this has also been the case for the basic RSP design. However, the decision variable can easily be changed from "more or less than 50% dead mice" to “the number of dead mice”. If none or only one of the three mice on the first design level dies, the dose for the next design level has to be increased, otherwise it is decreased. A similar procedure is used on the next design level. The simulation of this procedure in the present paper clearly demonstrated a substantial increase in the power of the RSP design. From the simulated results it seems that the increase in information in the model is so large that the fourth level of the design can be omitted. The number of animals can therefore be reduced from the initial 36 in the basic RSP design to 24 by including the *k*-adjustment dose factor and by increasing the number of animals at increasing design levels. The simulation results also indicated that the optimised 3-level RSP design estimated the LD_50_ as well as all the other 4-level RSP variants. By reducing the number of levels in the design from 4 to 3, only 15 animals are needed. The optimised RSP design created more pathways, estimated LD_50_ slightly higher and performed better than the other two RSP designs.

The RSP design should be performed sequentially and the dose to be used on the next design level is not available until the experiment on the previous level has ended. This may result increase the duration of the study as a whole. Furthermore, experimental conditions such as the weight of the animals, the composition of the test compound, variations in the time to death and environmental factors may differ between design levels and influence the results. A reduction from four to three design levels is therefore an advantage for many reasons.

The complete optimised RSP design was used in the AZA1 toxicity study. In this experiment, the predefined dose window of AZA1 was quite large and the dose used on the first design level proved to be far from the end result. The study demonstrated the power of the design and that the area of interest was already detected by the third design level. This underlines the fact that the fourth design level of the 9 mice was not needed and 15 mice would have been sufficient. If the AZA1 study had been performed using optimised 3-level RSP design, the k-adjustment factor would have been 1.78 and the estimated LD_50_ would not have been different from the value estimated by using the optimised 4-level RSP design. In order to simulate an optimal 3-level RSP for estimation of the LD_50_ of AZA1, the results obtained in the prospective 4-level study were used. All the three mice at the first design level died, which led to a dose of 88 μg/kg being assigned on the second level, where 3 of 5 mice died. The dose for the third design level was then reduced to 68 μg/kg and 3 of 7 mice died. This simulation estimated the LD_50_ of AZA1 to be 76.3 (95% CI: 38.5 - 101.58) and 75.7 μg/kg BW (95% CI 49.4 – 116.2) using isotonic regression and the Spearman-Karber method, respectively. The confidence intervals were, as expected, larger compared to those in the prospective 4-level RSP design study, but they still support the use of only 3 dose levels.

The simulated comparison between 3-level RSP, UDPs and RW design showed that the RSP reduced the number of study sequences compared to the other two designs. One way of comparing the information from the three designs is to compare the length of the CI for the parameter in question. If this is done, the RSP design was clearly superior to both the RW and the UDPs. Both the simulation and the results from the AZA1 study showed that the RSP designs rapidly converge on the area of interest. The RSP design was found to increase the information that could be obtained and it was possible to develop a dose–response curve.

The optimized 3-level RSP-design has recently been used in two other studies (manuscripts in preparation). However, further studies are needed to confirm the performance of the 3-level RSP design.

The dose to be used on a given design level in the RSP design is derived mathematically and may in some cases be impossible to titrate or administer in the laboratory. In these cases the dose must be adjusted to the nearest practicable value.

The determination of LD_50_ in the present paper was merely used to demonstrate how the RSP design was optimised. The designs can also be used to estimate the LD_X_, where 50% is replaced by x%. This design can also be used in other areas of medicine. The most relevant of these may be dose-finding studies, but the RSP design is also applicable to studies where the primary aim is estimation of quantile response.

## Conclusions

The RSP is a sequential design, which can reduce the number of animals needed to a minimum without loss of information. The design takes into account the variation in response between animals, using prior knowledge and information obtained during the study and it converges rapidly on the area of interest. It is as least as efficient as both the UDPs and RW designs.

## Competing interest

The authors declare that they have no competing interests.

## Author’s contributions

All the authors participated in development of the manuscript. The Response Surface Pathway design has mainly been developed by SD and SL who also performed the statistical analyses and wrote the initial draft of the manuscript. The laboratory work was performed or designed by AS, TA and JAAB. All the authors have participated in the literature review and development of the manuscript, and have approved the final version.

## Pre-publication history

The pre-publication history for this paper can be accessed here:

http://www.biomedcentral.com/2050-6511/15/18/prepub

## References

[B1] Seidle T Robinson S Holmes T Creton S Prieto P Scheel Chlebus M Cross-sector review of drivers and available 3Rs approaches for acute systemic toxicity testing Toxicol Sci 2010 116 382 396 10.1093/toxsci/kfq143 20484382 PMC2905404

[B2] Hess P Grune B Anderson DB Aune T Botana LM Caricato P Van Egmond HP Halder M Hall S Lawrence JF Moffat C Poletti R Richmond J Rossini GP Seamer C Vilageliu JS Three Rs Approaches in Marine Biotoxin Testing. The Report and Recommendations of a joint ECVAM/DG SANCO Workshop (ECVAM Workshop 54) Altern Lab Anim 2006 34 193 224 16704292 10.1177/026119290603400207

[B3] Alexander J Benford D Boobis A Ceccatelli S Cravedi J Domenico AD Doerge D Dogliotti E Edler L Farmer P Filipic M Fink-Gremmels J Fürst P Guerin T Knutsen HK Machala M Schlatter J Mutti A Schlatter J Leeuwen RV Verger P Opinion of the Scientific Committee/Scientific Panel, Marine biotoxins in shellfish - Summary on regulated marine biotoxins EFSA journal 2009 7 1 23

[B4] Barnes D Alford-Stevens A Birnbaum L Kutz FW Wood W Patton D Toxicity equivalency factors for PCBs? Qual Assur 1991 1 70 81 1669971

[B5] Barnes DG Toxicity equivalents and EPA's risk assessment of 2,3,7,8-TCDD Sci Total Environ 1991 104 73 86 10.1016/0048-9697(91)90008-3 1871591

[B6] Safe SH Comparative Toxicology and Mechanism of Action of Polychlorinated Dibenzo-P-Dioxins and Dibenzofurans Annu Rev Pharmacol Toxicol 1986 26 371 399 10.1146/annurev.pa.26.040186.002103 3013079

[B7] Aune T Sorby R Yasumoto T Ramstad H Landsverk T Comparison of oral and intraperitoneal toxicity of yessotoxin towards mice Toxicon 2002 40 77 82 10.1016/S0041-0101(01)00192-1 11602282

[B8] Rusche B The 3Rs and animal welfare - conflict or the way forward? ALTEX 2003 20 63 76 14671703

[B9] Rispin A Farrar D Margosches E Gupta K Stitzel K Carr G Greene M Meyer W McCall D Alternative methods for the median lethal dose (LD(50)) test: the up-and-down procedure for acute oral toxicity ILAR J 2002 43 233 243 10.1093/ilar.43.4.233 12391399

[B10] Lorke D A new approach to practical acute toxicity testing Arch Toxicol 1983 54 275 287 10.1007/BF01234480 6667118

[B11] Zbinden G Flury-Roversi M Significance of the LD50-test for the toxicological evaluation of chemical substances Arch Toxicol 1981 47 77 99 10.1007/BF00332351 7271444

[B12] Botham PA Acute systemic toxicity–prospects for tiered testing strategies Toxicol In Vitro 2004 18 227 230 10.1016/S0887-2333(03)00143-7 14757114

[B13] OECD Guideline for the testing of chemical 425, Acute oral toxicity- Up-and-Down Procedure (UDP) [http://www.oecd-ilibrary.org/content/book/9789264071049-en]

[B14] Dixon WJ Mood AM A Method for Obtaining and Analyzing Sensitivity Data J Am Stat Assoc 1948 43 109 126 10.1080/01621459.1948.10483254

[B15] Bruce RD An Up-and-Down Procedure for Acute Toxicity Testing Toxicol Sci 1985 5 151 157 10.1093/toxsci/5.1.151 3987991

[B16] Tsutakawa RK Random Walk Design in Bio-Assay J Am Stat Assoc 1967 62 842 856 10.1080/01621459.1967.10500897

[B17] Myer RH Montgomery DC Response Surface Methodology: Process and Product Optimization Using Designed Experiments 2002 New Jersey: John Wiley and sons

[B18] Aune T Larsen S Aasen JAB Rehmann N Satake M Hess P Relative toxicity of dinophysistoxin-2 (DTX-2) compared with okadaic acid, based on acute intraperitoneal toxicity in mice Toxicon 2007 49 1 7 10.1016/j.toxicon.2006.07.033 17092529

[B19] Aune T Aasen JAB Miles CO Larsen S Effect of mouse strain and gender on LD50 of yessotoxin Toxicon 2008 52 535 540 10.1016/j.toxicon.2008.06.025 18657566

[B20] Fisher G Series and Sequences 1996 Phoenix Education: Putney

[B21] Finney RL Thomas GB Calculus 1990 1 Reading: Addison-Wesley Publishing Company

[B22] Stylianou M Flournoy N Dose Finding Using the Biased Coin Up-and-Down Design and Isotonic Regression Biometrics 2002 58 171 177 10.1111/j.0006-341X.2002.00171.x 11890313

[B23] Hamilton MA Russo RC Thurston RV Trimmed Spearman-Karber method for estimating median lethal concentrations in toxicity bioassays Environ Sci Technol 1977 11 714 719 10.1021/es60130a004

[B24] Stylianou M Proschan M Flournoy N Estimating the probability of toxicity at the target dose following an up-and-down design Statist Med 2003 22 535 543 10.1002/sim.1351 12590412

[B25] Lethal Concentration, 50% (LC50) [http://sdi.odu.edu/model/lc50.php]

[B26] Acute Oral Toxicity (AOT) Up-And-Down-Procedure [http://www.epa.gov/oppfead1/harmonization/]

[B27] Silvapulle MJ On the Existence of Maximum Likelihood Estimators for the Binomial Response Models J R Stat Soc Series B Stat Methodol 1981 43 310 313

